# Clinical and diagnostic insights into brucellar arthritis: a single-center retrospective cohort study

**DOI:** 10.3389/fcimb.2025.1611398

**Published:** 2025-06-10

**Authors:** Qiangsheng Feng, Yuejuan Song, Yuan Xing, Xiaoqin Ha

**Affiliations:** Department of Clinical Laboratory, The 940th Hospital of Joint Logistics Support Force of People’s Liberation Army, Lanzhou, China

**Keywords:** brucellosis arthropathy, brucella melitensis, joint fluid-blood bottle culture, clinical characteristics, infection biomarker

## Abstract

**Background:**

This study evaluates the diagnostic value of etiological and serological testing in distinguishing brucellosis arthropathy from other inflammatory joint diseases.

**Methods:**

A retrospective analysis was conducted on 68 cases of brucellosis arthropathy diagnosed between 2012 and 2024, alongside 60 non-infected controls. Bacterial cultures were performed using blood, joint fluid-blood bottle culture, and joint tissue samples, with microbial identification via VITEK Compact-II or MALDI-TOF MS. Clinical features, serological results (Standard Agglutination Test [SAT] and Rose Bengal Test [RBT]), and imaging findings were analyzed. The diagnostic performance of biomarkers was assessed using receiver operating characteristic (ROC) curves.

**Results:**

Among the 68 cases, 22 (32.4%) were confirmed by bacterial culture, with Brucella melitensis identified as the causative agent. Joint fluid-blood bottle culture was the most effective method (62.2%), with a mean detection time of 74.8 ± 17.9 hours (range: 41–110 hours) in aerobic bottles. Blood culture and joint tissue culture yielded positive results in 40.9% and 4.5% of cases, respectively. Brucellosis arthropathy accounted for 7.5% of total brucellosis cases, predominantly affecting males (67.6%) with a median age of 43.1 ± 13.2 years. Brucellosis arthropathy infection median time were 90[30,343] days. The knee joint was the most commonly affected site (64.7%), followed by the hip (20.6%) and sacroiliac joints (10.3%). Imaging revealed septic arthritis (20.7%), joint effusion (31.0%), bone destruction (12.0%), degenerative changes (10.3%), and prosthetic joint infection (6.9%). The sensitivity for Brucella culture, SAT, and RBT were 69.7%, 87.7%, and 91.2%, respectively, with a combined sensitivity of 92.6%. ROC analysis identified CRP as a highly sensitive and specific biomarker (cutoff: 4.07 mg/mL; sensitivity: 84.2%, specificity: 72.2%; Z = 5.568, p < 0.001). All patients were treated with doxycycline and rifampicin for 3 months, with 34% requiring surgical intervention. The prognosis was satisfactory in all cases.

**Conclusions:**

Brucellosis arthropathy, often chronic and predominantly affecting the knee and hip joints, presents with septic arthritis, joint effusion, and bone destruction on imaging. Diagnosis can be effectively achieved through aerobic joint fluid-blood bottle culture, SAT, and RBT. Early diagnosis and combined medical-surgical management yield favorable outcomes.

## 1 Introduction

Brucellosis arthropathy, a common complication of brucellosis caused by Brucella species, is characterized by non-specific clinical manifestations that often mimic other inflammatory joint diseases, leading to frequent misdiagnosis even in endemic regions (McAllister, 1976). Studies report a prevalence of 2–45% for sacroiliac arthritis and 14–26% for peripheral arthritis among brucellosis patients (Jin et al., 2023). Despite its clinical significance, research on joint brucellosis remains limited, primarily confined to case reports (Stumpner et al., 2023; Ling et al., 2025; Jahmani et al., 2021; Pan et al., 2024). Laboratory diagnosis of brucellosis typically relies on aerobic blood culture, standard bacterial culture, Rose Bengal Test (RBT), and Standard Agglutination Test (SAT), complemented by epidemiological data (Qiangsheng et al., 2023).

This study focuses on improving the diagnosis of brucellosis arthropathy by utilizing joint fluid-blood bottle culture and blood culture methods, combined with SAT and RBT, to enhance diagnostic accuracy. Additionally, we analyze clinical features and infection biomarkers to distinguish brucellosis arthropathy from other joint disorders. Our findings are presented as follows:

## 2 Materials and methods

### 2.1 General materials

The study analyzed cases of brucellosis arthropathy recorded at the 940th Hospital in Lanzhou, China, between January 2012 and January 2024. The research was approved by the Scientific Research Management Ethics Committee (Approval No: 2022KYLL301). The positive group consisted of 68 patients, including 46 males and 22 females, with a male-to-female ratio of 2.09. The median age of the patients was 43.1 ± 13.2 years, ranging from 11 to 66 years old. Brucellosis arthropathy accounted for 7.5% of all brucellosis cases during this period. For comparison, a control group of 60 non-infection patients was selected from the same hospital. This group included 23 cases of arthromeningitis, 25 cases of osteoarthritis, 20 cases of rheumatoid arthritis, and 2 cases of gouty arthritis. The male-to-female ratio in the control group was 1.5, and the median age was 51.4 ± 21.9 years.

#### 2.1.1 Blood cultures and joint fluid-blood bottle culture

In patients with clinically suspected infectious arthritis, blood or joint fluid (10–20 mL) was collected and injected into both anaerobic and aerobic blood culture bottles. The blood culture bottles used were either BacT/ALERT (bioMérieux, Inc., Durham, NC) aerobic FA and anaerobic SN bottles or BD (Becton, Dickinson, and Company) Plus aerobic and anaerobic bottles. These were incubated in the BacT/ALERT 3D (bioMérieux, Inc.) or BACTEC™ FX200 (Becton, Dickinson, and Company) automated monitoring systems, respectively, for 7 days in the hospital’s clinical microbiology laboratory. When the aerobic bottle triggered a positive alarm within 2–4 days, characterized by an “S”-shaped growth curve, a sterile syringe was used to extract the culture medium from the bottle. Direct Gram staining and Swiss-Giemsa staining were performed immediately, and the sample was simultaneously inoculated onto a blood plate for general bacterial culture. The culture was incubated at 35°C under normal atmospheric conditions for 72 hours or longer. If Gram-negative small bacteria were observed under the microscope and the Swiss-Giemsa stain revealed fine sand-like or clump-like clusters, a preliminary oral report was immediately communicated to the clinical team, suggesting a suspected Brucella infection. Once colonies formed, microbial identification was performed using the VITEK Compact-II automatic microorganism identification system with a GN Card (bioMérieux, Inc., Durham, NC) or MALDI-TOF MS. The results were reported to the clinical departments via the LIS system. For patients who underwent blood culture testing two or more times, only one instance was recorded and counted to avoid duplication. This standardized protocol ensured accurate and timely identification of Brucella and other pathogens in suspected infectious arthritis cases. All specimens and cultures were autoclaved at 121°C for 30 minutes and transported out of the laboratory.

#### 2.1.2 Joint tissue culture

When aseptically collected joint tissue samples were obtained during clinical operations, tissue blocks measuring 2–6 mm³ were selected and inoculated onto a blood plate. The general bacterial culture was then incubated at 35°C under normal atmospheric conditions for at least 72 hours. After colony formation, microbial identification was performed using the corresponding GN card on the VITEK Compact-II automatic microorganism identification system or MALDI-TOF MS. All procedures were conducted in a biological safety cabinet to ensure aseptic conditions, and all specimens and cultures were autoclaved at 121°C for 30 minutes and transported out of the laboratory. The identification results were reported to the clinical departments through the LIS system. This protocol ensured accurate and safe processing of joint tissue samples for pathogen identification.

### 2.2 Serum inflammatory biomarker detection in patients with brucellosis

For the initial admission test, the infection biomarkers were prioritized. Serum samples for procalcitonin (PCT) and C-reactive protein (CRP) were collected in dry tubes with gel separators and centrifuged within the first 2 hours. PCT levels were measured using the E-170 automatic analyzer (Roche), with a cut-off value of 0.046 ng/mL. CRP levels were quantified using an immunoturbidimetric assay on the ARCHITECT c-System (Abbott Laboratories, IL, USA), with a detection limit of 0.5 mg/dL and an imprecision of ≤ 5% total coefficient of variation, as specified by the manufacturer. Synovial fluid samples were collected in EDTA-K2 anticoagulant tubes and analyzed within 2 hours of collection for white blood cell (WBC) count and neutrophil percentage (NEU%) using an automated hematology analyzer (Minray, China). For prevent data duplication, only one set of infection biomarker results per patient was included. Additionally, 60 non-infection patients from the hospital were selected as the control group for comparison of infection biomarkers. This approach ensured a standardized and accurate assessment of infection biomarkers in both brucellosis and control groups.

### 2.3 Serological testing methods: RBT and SAT

The Standard Tube Agglutination Test (SAT) and Rose Bengal Test (RBT) antigens were obtained from the Institute of Infectious Diseases, China Center for Disease Control and Prevention. For the RBT, a card agglutination method was used. A total of 0.03 mL of the tested serum was mixed with 0.03 mL of the antigen, and the results were observed within 4 minutes. A reaction above “+” was considered positive. For the SAT, serial 2-fold dilutions of the patient’s serum (ranging from 1:12.5 to 1:400) were mixed with Brucella antigen in the wells of a microtiter plate. The plate was incubated at (37 ± 1) °C for 24 hours. After incubation, the results were compared with a turbidimetric control tube. A titer of ≥1:100 (++) was considered diagnostic. For patients with a disease course of more than one year, a titer of ≥1:50 (++) was also considered diagnostic.

### 2.4 Clinical diagnostic standard for brucellosis

Clinical diagnosis based on Brucella culture positive, epidemiological history, and laboratory test results.

#### 2.4.1 Epidemiological history

The patient had a history of close contact with livestock or livestock products suspected of Brucella infection before the onset of symptoms. Brucella can be transmitted through contact with animal tissues, blood, vaginal secretions, aborted fetuses, and especially placentae. This exposure history is a critical factor in assessing the likelihood of brucellosis infection.

#### 2.4.2 Clinical suspect case definitions

Cases that are suspected and have a positive SAT titer (but not a positive culture), Standard Tube Agglutination Test (SAT) titer of ≥1:100 (++), or/and For patients with a disease course of more than one year, an SAT titer of ≥1:50 (++).

#### 2.4.3 A confirmed case of brucellosis is defined

Confirmed for those cases in which the pathogen has been isolated from blood or joint fluid/tissue.

#### 2.4.4 Chronic brucellar arthritis cases were defined

Chronic brucellar arthritis cases were defined as patients presenting with persistent joint pain and functional limitations (e.g., restricted range of motion) for ≥6 months, supported by laboratory confirmation of Brucella infection through microbiological culture, or serological testing (e.g., SAT ≥1:160), and exclusion of alternative inflammatory or degenerative joint pathologies.

### 2.5 Statistics

Statistical analysis was performed using SPSS 22.0 software. For measurement data (e.g., age, PCT, CRP, WBC, and NEU%), variables with a normal distribution were expressed as median (M) with interquartile range (P25, P75). The levels of PCT and CRP, which were non-normally distributed, were also expressed as M (P25, P75), and the Mann-Whitney U test was used for comparison. For enumeration data (e.g., gender), results were expressed as rates, and the χ² test was applied. A p-value < 0.001 was considered statistically significant. The general information comparing the infection group and control group is summarized in **Table 1**. Additionally, the sensitivity and specificity of clinical infection biomarkers in the infection and control groups were analyzed using the receiver operating characteristic (ROC) curve, with the area under the ROC curve (AUC) calculated to evaluate diagnostic performance.

**Table 1:** General information on the infection and control group.

Group	Case	Men/women	Age	PCT	CRP	Joint fluid -WBC	Joint fluid -NEU%
		(case)	(x ± s, year)	[M(P25, P75), ng/ml]	[M(P25, P75),mg/L]	[M(P25, P75),× 10^6^/L)	[M(P25, P75)
Infection	68	46/22	43.1± 13.2	0.07 [0.03,0.10]	4.01 [0.80,6.45]	7000 [2650,14400]	68 [55,85]
Control	60	36/24	51.4 ± 21.9	0.18 [0.05,0.33]	1.19 [0.15,0.46]	2787 [393,10883]	44 [15,77]
T		χ^2^=0.81	t=1.034	Z=-3.46	Z=5.57	Z =1.65	Z =1.69
P		0.368	0.303	0.001	<0.001	0.10	0.09

*P < 0.01* was considered statistically significant.

## 3 Result

### 3.1 Clinical characteristics analysis of human brucellosis arthropathy

**Figure 1:**
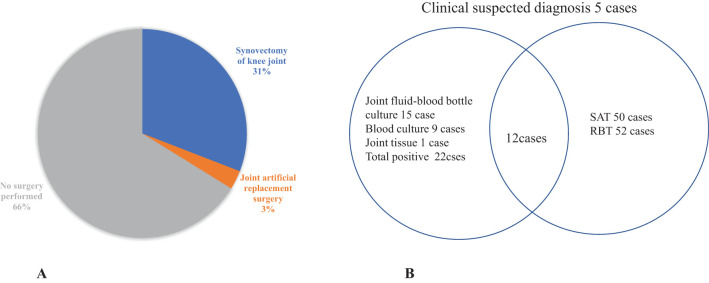
**(A)** Brucellosis arthritis surgery. These 68 cases of joint brucellosis were treated with doxycycline and rifampicin for 3 months, of which 34% were treated by surgery, and the prognosis is satisfactory. **(B)** Brucellosis arthritis diagnostic method: SAT: Standard agglutination test RBT: Rose Bengal test; SAT sensitivity:87.7% (50/57), RBT sensitivity: 91.2% (52/57); Culture sensitivity 66.7% (22/33), with a combined sensitivity of 92.6%.

In this study, the median age of patients with human brucellosis arthropathy was 43.1 ± 13.2 years, with an age range of 11 to 66 years. The male-to-female ratio was 2.09, and brucellosis arthropathy accounted for 7.5% of the total brucellosis cases in the hospital. Among the 68 cases of joint brucellosis, all patients were treated with a combination of doxycycline and rifampicin for 3 months. Additionally, 34% of these patients underwent surgical treatment. The prognosis was satisfactory, as illustrated in **Figure 1A**.

### 3.2 Brucellosis arthropathy diagnostic method

Data from 68 cases of brucellosis in our hospital from 2012 to 2024 were analyzed. Among these, 23 cases were culture-positive and identified as Brucella melitensis using automated instruments. Joint fluid-blood bottle culture was the primary diagnostic method in 15 cases (62.2%,15/23), with all positive cultures detected in aerobic bottles within an average time of 74.8 ± 17.9 hours (n=22 bottles), ranging from 41 to 110 hours. Blood culture and joint tissue culture accounted for 40.9% and 4.5% of the cases, respectively. The diagnostic methods and their sensitivity were as follows: Brucella spp. Culture: 69.7% (23/33). Standard Tube Agglutination Test (SAT): 87.7% (50/57), Rose Bengal Test (RBT): 91.2% (52/57). The distribution of diagnostic approaches was: Pathogenic diagnosis (culture): 33.8% (23/68), Serological diagnosis (SAT/RBT): 55.8% (40/68), Clinically suspected diagnosis: 7.4% (5/68). The sensitivity of combining all three detection methods (culture, SAT, and RBT) was 2.6% (63/68), as illustrated in **Figure 1B**.

### 3.3 Brucellosis arthropathy infected joint and image display

**Figure 2:**
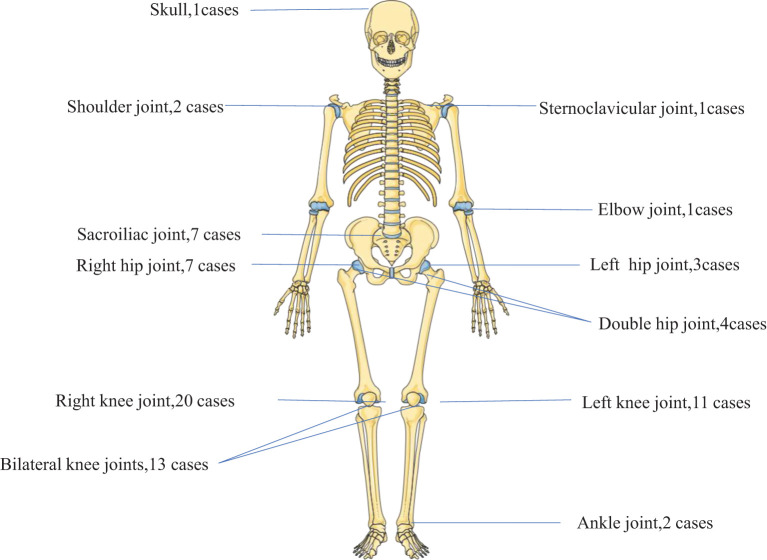
Brucellosis arthritis (n= 68 cases). Brucellosis arthropathy mainly affected the knee joint (64.7%), followed by the hip joint (20.6%), the sacroiliac joint (10.3%) and other joints (11.1%), whereas multi-joint infection occurred in 7.4%.

In cases of brucellosis arthropathy, the main site of infection was the knee joint, accounting for 64.7% of cases. This was followed by the hip joint (20.6%), sacroiliac joint (10.3%), and other joints (11.1%). Multi-joint infection was observed in 7.4% of cases, as illustrated in **Figure 2**. In 58 cases joint image display septic arthritis at 20.7%, joint effusion at 31.0%, bone destruction at 12%, degenerative changes at 10.3%, normal at 10.3%, and PJI (prosthetic joint infection) at 6.9%.

### 3.4 Chronic Brucellosis arthropathy infection

**Figure 3:**
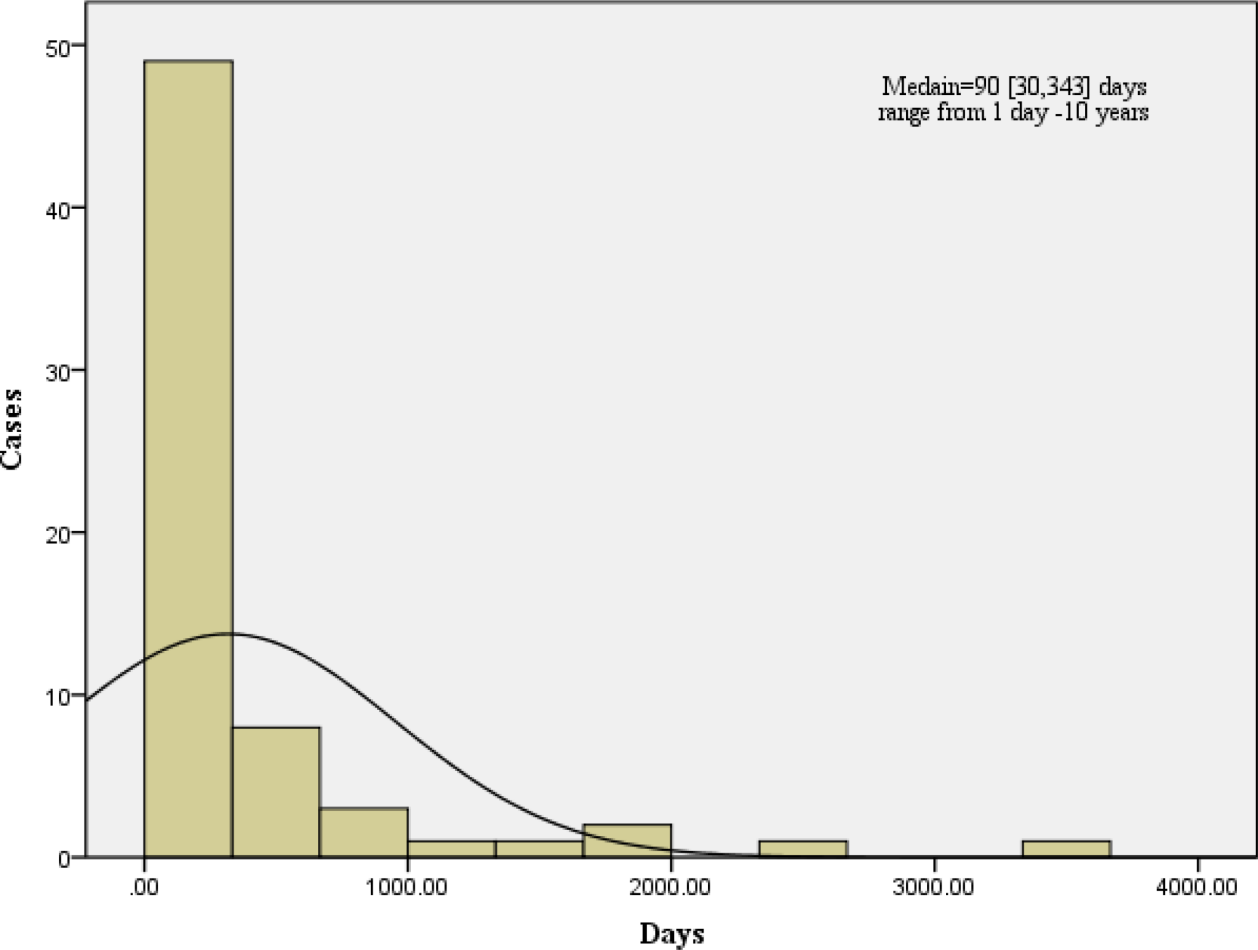
Brucellosis arthropathy infection time(days). Brucellosis arthropathy and the Brucellosis arthropathy infection median time were 90[30,343] days, ranging from 2 days to 10 years.

Between 2012 and 2024, 68 cases of brucellosis-associated arthropathy were documented with a median infection duration of 90 days (IQR 30-343). Within this cohort, 19 cases (27.9%) progressed to chronic brucellar arthritis, defined by persistent joint pain and functional impairment (including restricted range of motion) lasting ≥6 months. These chronic cases demonstrated substantially prolonged disease courses, with a median duration of 540 days (IQR 365-1460) and clinical manifestations persisting from 6 months to 10 years. The temporal progression patterns of these cases are visually summarized in **Figure 3**.

### 3.5 ROC curve analysis of clinical biomarkers in brucellosis and control group

**Figure 4:**
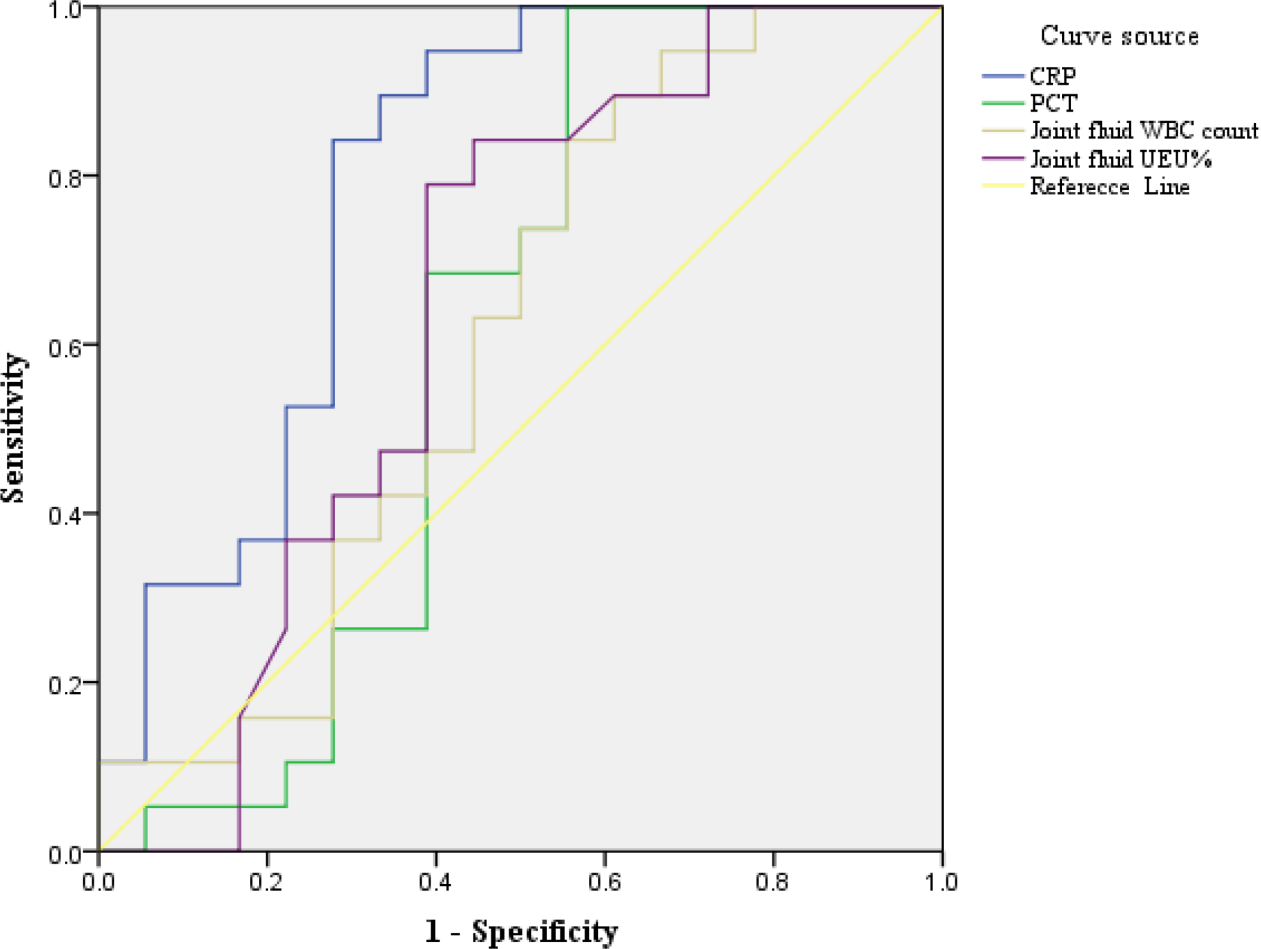
ROC curve analysis of clinical biomarkers in Brucellosis arthritis infection. ROC curve analysis of biomarkers in patients with brucellosis and non-brucellosis. CRP- C-reactive protein, PCT – C-reactive protein, NEU%-Percentage of neutrophils, WBC-white blood cell. The AUC method was employed to evaluate the biomarker for the diagnostic value of brucellosis showing that joint fluid -WBC, joint fluid -NEU%, PCT, and CRP were 0.605, 0.645, 0.605, and 0.792 respectively confidence in 95% intervals as shown in **Table 2**.

The discriminatory ability for brucellosis as determined by the AUC-ROC for each biomarker is presented in **Figure 4**. For overall biomarkers, the AUC-ROC value for CRP was 0.792, and when the cut-off value of 4.07 mg/ml, the sensitivity and specificity were 0.842 and 0.722 respectively, and the U test also indicated CRP had a significant difference, Z=5.568, *p*<0.001 presented in **Table 1** and **Table 2**. And there were no significant differences regarding age, sex Joint fluid -WBC and, joint fluid -NEU% distribution between the Infection group and the Control group.

**Table 2:** AUC method employed to evaluate the biomarker for the diagnostic value of brucellosis.

Biomarkers	AUC value	cut off value	sensitivity(%)	specificity(%)	Asymptotic 95% Confidence interval	
					Lower bound	Upper bound
Joint fluid -NUE%	0.645	–	-	-	0.455	0.835
Joint fluid -WBC	0.605	–	–	–	0.318	0.453
PCT	0.605	–	-	-	0.405	0.806
CRP	0.792	4.07 mg/ml	0.842	0,722	0.639	0.946

The test result variable(s): Joint fluid -NEU, Joint fluid -WBC, CRP, PCT has at least one tie between the positive actual state group and the negative actual state group. Statistics may be biased.

## 4 Discussion

Brucellosis is a zoonotic disease that causes systemic symptoms and can affect multiple organs and tissues. In our study, the median age of patients with human brucellosis arthropathy was 43.1 ± 13.2 years, and brucellosis arthropathy accounted for 7.5% of total brucellosis cases. This rate is lower than the 14-26% prevalence of peripheral arthritis reported by Unuvar GK (Unuvar et al., 2019). In our hospital, brucellosis arthropathy was treated with a combination of doxycycline and rifampicin for 3 months, and the prognosis was satisfactory, consistent with findings from Bukkems (Bosilkovski et al., 2016) and Zhou P (Zhou et al., 2023).

Initial symptoms of hip arthritis are often subtle, making diagnosis and treatment challenging (McAllister, 1976). Blood culture, the traditional method for diagnosing Brucella spp., has limitations due to the bacteria’s long generation time and low isolation rates (Franco et al., 2007; Yagupsky et al., 2019). In this study, synovial fluid-blood culture bottles served as the principal diagnostic modality for etiological confirmation, constituting 62.2% of confirmed diagnoses. All positive cultures were exclusively identified in aerobic bottles, with microbial growth detected after a mean incubation period of 74.8 ± 17.9 hours (range: 41–110 hours). Notably, this investigation demonstrates that extending the incubation duration of synovial fluid cultures to a minimum of 110 hours (4.6 days) is critical when brucellosis-associated arthropathy is clinically suspected – a key operational recommendation derived from our empirical observations. The sensitivity for diagnostic methods were: Brucella spp. Culture: 69.7%, Standard Tube Agglutination Test (SAT): 87.7%, Rose Bengal Test (RBT): 91.2%. The sensitivity of combining these three methods was 92.6%, a finding rarely reported in the literature.

Bone and joint infections are among the most common complications of human brucellosis, affecting 10-85% of patients (Esmaeilnejad-Ganji and Esmaeilnejad-Ganji, 2019; Tajerian et al., 2024). In our study, brucellosis arthropathy primarily affected the knee joint (64.7%), followed by the hip joint (20.6%), sacroiliac joint (10.3%), and other joints (11.1%). Multi-joint infection was observed in 7.4% of cases. While several cases of knee joint brucellosis have been reported (Al Hariri et al., 2022; Wang and Zhang, 2022; Hassan et al., 2024), cases involving other joints are rare. Prosthetic joint infection (PJI) accounts for 6.9% of cases and is prone to misdiagnosis.

Bone scans and MRIs can help avoid misdiagnosis and are widely used in clinical practice (Dayan et al., 2009). In our study, 58 cases of joint image display septic arthritis at 20.7%, joint effusion at 31.0%, bone destruction at 12%, degenerative changes at 10.3%, normal at 10.3%. Of note, there are few literature reports on the imaging manifestations of brucellosis arthropathy. In a young patient with multifocal brucellosis, magnetic resonance imaging (MRI) of the knee revealed concurrent joint effusion and osteomyelitis (Dahani et al., 2025).

Chronic brucellosis arthropathy refers to long-term brucellosis, caused by bacteria of the genus Brucella. The CDC and the WHO do not precisely define chronic brucellosis. Generally, symptoms persist for over a year after the initial diagnosis (Qureshi et al., 2023). We define chronic brucellosis arthropathy infection cases: as patients with continuous joint pain and limited motion and symptoms prolonging with progressive, and it was diagnosed by etiology and serology after hospitalization. In our study, the 68 brucellosis arthropathy infection median time was 90 [30,343] days, ranging from 2 days to 10 years. A case report of shoulder arthroplasty after chronic brucellosis of glenohumeral joint septic arthritis for two years (Chernchujit et al., 2022).

The discriminatory ability for brucellosis was assessed using the AUC-ROC for each biomarker. For CRP, the AUC-ROC value was 0.792. At a cut-off value of 4.07 mg/mL, the sensitivity and specificity were 0.842 and 0.722, respectively. The Mann-Whitney U test confirmed a significant difference (Z=5.568, p<0.001). Same to Akya A reported (Akya et al., 2020) serum CRP can be used as valuable markers in the preliminary diagnosis of brucellosis. But it may be interesting to comment that inflammatory markers (especially TNF-α, IL-8 and MCP-1) were also differentially increased in the synovial fluid of a patient with Brucella bursitis as compared to samples from rheumatoid arthritis or septic arthritis (Wallach et al., 2010).

Our findings highlight the importance of joint fluid-blood bottle culture with extended incubation periods, combined diagnostic methods, and imaging techniques for accurate diagnosis and management of brucellosis arthropathy. The knee joint is the most commonly affected site, and CRP is a valuable biomarker for differentiating brucellosis cases. These insights contribute to improving the diagnosis and treatment of this challenging condition.

### 4.1 Limitations

We found that joint fluid-blood bottle culture, a key diagnostic method for brucellosis arthropathy, has not been widely adopted due to limited sample size in clinical practice. In our hospital, we have reported cases of chronic brucellosis arthropathy; however, the latent infection rate of brucellosis arthropathy has not been systematically calculated. Additionally, due to biosafety concerns, routine antimicrobial susceptibility testing for *Brucella* spp. is not feasible in our setting.

### 4.2 Application

Our findings demonstrate that joint fluid-blood bottle culture is an effective method for culturing brucellosis arthropathy specimens, significantly improving the positive rate of etiological diagnosis. Given its diagnostic efficacy, this technology warrants wider application in clinical practice.

## Data Availability

The raw data supporting the conclusions of this article will be made available by the authors, without undue reservation.
